# Pediatrician’s role in vaccinating children and families for COVID-19: no one left behind

**DOI:** 10.1038/s41390-021-01804-z

**Published:** 2021-11-24

**Authors:** Annabelle de St. Maurice, Tina L. Cheng, Sherin U. Devaskar, on behalf of the Pediatric Policy Council, Shetal Shah, Jean L. Raphael, Mona Patel, Jonathan Davis, DeWayne Pursley, Tina L. Cheng, Sherin U. Devaskar, Joyce Javier, Lois Lee, Lisa Robinson, Mary Leonard, Shale Wong, Beth Tarini, Monika Goyal

**Affiliations:** 1 Department of Pediatrics, David Geffen School of Medicine at UCLA, UCLA Mattel Children’s Hospital, Los Angeles, CA USA; 2 Department of Pediatrics, Cincinnati Children’s Hospital and University of Cincinnati, Cincinnati, OH USA; 3 New York Medical College, Valhalla, NY USA; 4 Baylor College of Medicine, Houston, TX USA; 5 University of Southern California, Los Angeles, CA USA; 6 Tufts Medical Center, Boston, MA USA; 7 Harvard Medical School, Boston, MA USA; 8 University of Cincinnati College of Medicine, Cincinnati, OH USA; 9 University of California Los Angeles, Los Angeles, CA USA; 10 University of Toronto Temerty Faculty of Medicine, Toronto, ON Canada; 11 Stanford University School of Medicine, Stanford, CA USA; 12 University of Colorado School of Medicine, Aurora, CO USA; 13 George Washington University School of Medicine and Health Sciences, Washington, DC USA

The importance of coronavirus disease 2019 (COVID-19) vaccines in children has been debated during the pandemic because the incidence of COVID-19 in children is lower than in adults, with particularly low rates in children <5 years of age.^1^ However, the physical and mental health of children has been greatly impacted by both direct and indirect effects of the COVID-19 pandemic. More than six million children have been diagnosed with COVID-19 in the United States alone^1^, over 4,000 children have been hospitalized^2^ and over 600 children have died.^1^ Globally, there have been over ten million COVID-19 cases and over 4000 deaths in persons 19 years of age and younger.^3^ The number of pediatric COVID-19 cases may be underestimated because children tend to have milder symptoms from infection and may be less likely to be tested than adults, particularly in low- and middle-income countries where access to testing may be limited. and COVID-19 case data are not always reported by age group.  Children infected with severe acute respiratory syndrome coronavirus 2 (SARS-CoV-2) are at risk of postinfectious complications including Multi-system Inflammatory Syndrome in Children^4^ and “long-COVID.”^5^ Pandemic mitigation measures such as school closures and cancellation of athletic activities have been associated with increased mental health difficulties and obesity rates in children, and have widened health disparities related to race/ethnicity and socioeconomic status.^6^ Beyond COVID-19 infection, the impact of the pandemic on children’s mental and physical wellbeing and educational progress has been far-reaching.^7^

More recently, the emergence of the more transmissible Delta variant has magnified the threat to children globally. The proportion of new COVID-19 cases in children has increased substantially from May through November  2021.^3^ During the week of November 11 2021, children accounted for over 120,000 new COVID-19 cases in the United States and represented 27% of the weekly COVID-19 cases.^8^ Until a greater proportion of the overall global population is vaccinated, new variants will continue to emerge.

For these reasons and more, it is important for all children aged 6 months to 17 years of age to be vaccinated against COVID-19 when vaccine efficacy and safety data are available and rigorously evaluated. Children constitute over 30% of the world’s population,^9^ yet are largely unvaccinated against COVID-19, because until recently the vaccine had not been approved for children <12 years of age. Childhood vaccinations have lagged primarily because COVID-19 vaccines were first studied in adults, given the higher incidence and severity of illness in the older population. In the United States, the only vaccine to have received emergency use authorization for children as young as 5 years of age is the Pfizer-BioNTech vaccine^10,11^ and several vaccine manufacturers are studying COVID-19 vaccines in children as young as 6 months of age. Fortunately, pediatric COVID-19 vaccinations have been found to be safe and effective for children 5 years of age and older,^12–14^ with the benefits far outweighing risks.

In addition, children play a role in COVID-19 transmission to other children and adults including the most vulnerable immunocompromised individuals. As Pratico and Ruggieri state in their article, unvaccinated children could potentially lead to transmission events in adults, similar to prior measles outbreaks.^15^ There are reports of infected children who have been vectors of transmission to their vaccinated or unvaccinated parents and this risk will likely increase as new variants arise and schools reopen.^16^ Although it is unlikely that COVID-19 will be eradicated given the high transmissibility of the virus and ability to mutate, vaccinating children can have a large impact on reducing transmission and severity of illness in children and adults while contributing to herd immunity. Pediatricians and other pediatric providers must play an active role in educating and providing vaccines to children as parents frequently cite their pediatric clinician as a trusted source of information regarding vaccines.^17^ Providing COVID-19 vaccines during well-child checks and sick visits may increase access to vaccines and ultimately increase vaccination rates in children. Pediatric practices are also an opportune location to offer vaccination to family members for multiple reasons, including: (1) infection transmission often occurs in families and vaccinating the entire family protects everyone; (2) pediatric clinicians are experts in discussing vaccines and vaccine hesitancy and are trusted; and (3) family members accompany children to pediatric visits where vaccines are available. Many practices have offered this approach as an efficient and effective mechanism to increase education and access to vaccines in their community. This is important as we know that the health of children is interdependent on the health of the family and their community. Finally, there also exists the opportunity to partner with schools, other educational institutions, and Pediatric-focused organizations to advocate for vaccines while educating families on the importance of vaccinating children of all ages when the vaccines are authorized for such use.

Researchers should continue to enroll young children in clinical trials aimed at evaluating the safety and efficacy of these vaccines. Vaccines should not be given to very young children until the efficacy, safety, and dosing have been thoroughly studied and vaccine experts have provided recommendations for administration.^18^ Potential rare adverse events, such as myocarditis,^19^ should continue to be investigated thoroughly. The background rate of myocarditis in individuals in the United States is estimated to be 1 to 10 per 100,000 persons annually with variations by age group and gender^20^ and the risk increases after SARS-CoV-2 infection. The rate of myocarditis after SARS-CoV-2 infection is estimated to be 6–34 times greater than the risk of messenger RNA vaccine-associated myocarditis and vaccine experts largely agree that the benefits of vaccination far outweigh the risk of myocarditis in children age 12 years and above.^21,22^ Similar risk–benefit analyses should be conducted on children who are younger than 12 years of age and maybe at lower risk of severe COVID-19 infection.

Global organizations including the World Health Organization and COVAX should work with vaccine manufacturers to expeditiously facilitate vaccine trials in children globally. Emergency-use authorization and licensure of these vaccines globally should be held to the same standards as adult vaccines. In addition to children, it is essential that future clinical trials include pregnant and lactating mothers to establish safety and efficacy in this vulnerable population (also protecting the fetus and other children). High-risk conditions such as preterm labor and pre-eclampsia-like features have emerged in mothers infected with SARS-CoV-2 and their infants have suffered the consequences of prematurity and fetal inflammatory response syndrome.^23^ Since more data have emerged demonstrating the safety and efficacy of these vaccines in pregnant and lactating women, pediatric organizations should support the Society for Maternal Fetal Medicine and ACOG^18^ in encouraging all pregnant and lactating women to receive a COVID-19 vaccination. Pediatric practices should also consider providing COVID-19 vaccines to parents.

Access to pediatric vaccines must be equitable globally. Thus far, the disparities in vaccine distribution globally have been striking. High-income nations have vaccinated the majority of their population, including the United Arab Emirates, which has vaccinated 98% of their population.^24^ In contrast, low- and middle-income countries such as Haiti have yet to vaccinate 1% of their population.^24^ While some wealthy nations are providing supplemental and booster doses to their population, many healthcare workers in low-income countries have yet to receive a single vaccine dose. It is important for leaders of wealthy nations to improve vaccine equity outside their own borders. Administering the primary COVID-19 vaccine series to high-risk individuals in low- and middle-income countries should be a global priority and responsibility of all nations. Public health leaders should balance priorities in rolling out vaccines to low-risk children age <5 vs the possibility of a substantial impact on global vaccine availability.

If adults and children in any global community are left behind in COVID-19 vaccine programs, it will be more difficult to mitigate the spread of SARS-CoV-2 for all, and pediatric hospitalizations and deaths will continue to increase globally.
